# Supplementary material for: WordCommentsAnalyzer: A windows software tool for qualitative research
Source: F1000Res. 2018 Sep 4;7:536. Originally published 2018 May 3. [Version 2] doi: 10.12688/f1000research.14819.2 (PMC6137411; doi:10.12688/f1000research.14819.2)
Supplement: Supplementary file 3 [file f1000research-7-17614-s0002.tgz › b2a79496-4ada-47c4-9682-a99ce867836d.zip!/Eight Iranian Health Journals/Journal of Knowledge and Health_25.docx]

© Journal of Knowledge and Health. This work is licensed under CC BY 4.0 (https://creativecommons.org/licenses/by/4.0/).

Volume & Issue: دوره 7، شماره 1: 1391

Title: اثر یک وهله فعالیت مقاومتي بر غلظت ادراری 8 ـ هیدروکسی ـ2ـ دی‌آکسی گوانوزین (8-OHdG) در ورزشکاران و غیرورزشکاران

http://knh.shmu.ac.ir/index.php/site/article/view/69

مقدمه: رادیکال‌های آزاد و گونه فعال اکسیژن در‌نتیجه متابولیسم طبیعی در سلول‌ها به‌طور مداوم تولید می‌شوند و تحت شرایط استرس سایکولوژیکی و جسمی افزایش می‌یابند. «استرس اکسیداتیو» شرایطی است که در آن تولید گونه فعال اکسیژن فراتر از ظرفیت سیستم آنتی‌اکسیدانی به‌منظور خنثی‌سازی این پراکسیدان‌ها باشد. در این شرایط پروتئین‌ها، لیپیدها و اسیدهای نوکليیک آسیب می‌بینند. بنابراین، هدف از این پژوهش بررسی اثر یک وهله فعالیت مقاومتی با شدت 80% یک تکرار بیشینه، بر بیومارکر آسیب اکسیداتیو DNA (8- هيدروكسي-2- دي اكسي گوانوزين) می‌باشد. مواد و روش‌ها: در این پژوهش نیمه‌تجربی 9 ورزشکار پرورش اندام و 9 فرد غیرورزشکار به‌صورت تصادفی انتخاب شدند. همه آزمودنی‌ها یک وهله فعالیت مقاومتی شامل 4 ست پرس سینه، پا، سر شانه، جلو بازو و لت را با شدت 80% یک تکرار بیشینه انجام دادند. نمونه‌گیری خون قبل و بلافاصله پس از فعالیت مقاومتی و نمونه‌های ادرار قبل، بلافاصله، 3 ساعت و 24 ساعت بعد از فعالیت مقاومتی در هر دو گروه جمع‌آوری گردید و جهت تعیین غلظت لاکتات و 8-OHdG استفاده شد. نتایج: غلظت 8-OHdG ادراری، بیومارکر آسیب اکسیداتیو DNA، بلافاصله و 24 ساعت پس از فعالیت مقاومتی در ورزشکاران به‌طور معناداری پايین‌تر از غیرورزشکاران بود (05/0P<). نتایج درون‌گروهی نشان داد که غلظت 8-OHdG ادرار غيرورزشكاران بلافاصله، 3 ساعت و 24 ساعت بعد از فعالیت مقاومتی شدید نسبت‌به قبل از فعالیت به‌طور معناداری بالاتر بود (05/0P<)؛ اما در ورزشکاران پرورش اندام فقط 24 ساعت پس از فعالیت مقاومتی افزایش معنادار در غلظت 8-OHdG ادرار مشاهده شد (05/0P<). نتیجه‌گیری: نتایج نشان داد که آسیب اکسیداتیو DNA در ورزشکاران پرورش اندام نسبت‌به غیرورزشکاران کمتر می‌باشد. این امر ممکن است به‌دلیل سابقه تمرینات منظم مقاومتی در ورزشکاران پرورش اندام باشد و امکان دارد که ظرفیت آنتی‌اکسیدانی در ورزشکاران به‌دلیل تمرینات منظم ورزشی تقویت شده باشد.

Title: بررسی تأثير برنامه آموزشي پيشگيري از استئوپروز بر آگاهي زنان کارمند

http://knh.shmu.ac.ir/index.php/site/article/view/72

مقدمه: استئوپروز شایع‌ترین بیماری متابولیک استخوان می‌باشد. در جمعیت زنان ایرانی، شیوع استئوپروز در ناحیه ستون فقرات و فمور 9/18% تخمین‌زده شده است. پژوهش حاضر با هدف بررسی تأثير برنامه آموزشی پیشگیری از استئوپروز بر ميزان آگاهي در زنان کارمند شهرستان شاهرود انجام شده است. مواد و روش‌ها: این مطالعۀ نيمه‌تجربي ازطریق پرسش‌نامه پایا و روا بین 180 خانم کارمند با مدرک تحصیلی ديپلم يا بالاتر که ازطريق دفتر امور بانوان جهت شركت در تحقيق معرفي شده بودند، طراحي و اجرا شد. پس از انجام آزمون اوليه و تجزيه‌و‌تحليل اطلاعات، برنامه آموزشي به شكل پمفلت، پوستر و سخنراني همراه با نمايش اسلايد توسط 3 متخصص زنان، پاتولوژي‌، داخلي در يك روز اجرا گرديد و آزمون ثانويه پنج هفته پس از آموزش انجام شد. تجزیه‌‌وتحلیل‌ داده‌ها با آزمون t و مک نمار انجام گردید. سطح معناداری برای تمام آزمون‌ها 05/0 در نظر گرفته شد. نتایج: نتایج پژوهش دال بر وجود اختلاف معنادار بین ميانگين امتیاز آگاهي درزمینه بيماري‌هاي مسبب استئوپروز، داروهاي ايجادكننده، داروهاي پيشگيري‌كننده، عوامل پيشگيري‌كننده، عوامل مساعدكننده و قابل‌اجتناب و عوامل مساعدكننده و غير‌قابل‌اجتناب استئوپروز قبل و بعد از آزمون بود (001/0P<). نتیجه‌گیری: باتوجه به میزان پایین آگاهی زنان کارمند درخصوص استئوپروز قبل از آموزش، ضرورت افزایش آگاهی جهت پیشگیری از این بیماری و حفظ سلامتی، افزایش کیفیت زندگی و استقلال در دوران سالمندی ضروری به‌نظر می‌رسد.

Title: ارزیابی تغذیه‌ای دختران نوجوان مدارس شهر شاهرود

http://knh.shmu.ac.ir/index.php/site/article/view/67

مقدمه: نوجواني يكي از بحراني‌ترين دوره‌هاي رشد انسان محسوب می‌شود که تغذیه نقش بسیار مؤثری در آن دارد. هدف از این مطالعه ارزیابی تغذیه‌ای دختران نوجوان مدارس راهنمایی شهر شاهرود می‌باشد. مواد و روش‌ها: در اين مطالعه مقطعي، تعداد 418 دانش‌آموز دختر مقطع راهنمايي به‌روش نمونه‌گيري خوشه‌اي دو مرحله‌اي انتخاب و داده‌ها براساس مصاحبه و اندازه‌گيري شاخص‌هاي تن‌سنجي جمع‌آوري گرديد. ابزار جمع‌آوری اطلاعات شامل پرسش‌نامۀ مشخصات دموگرافيك، يادآمد خوراك 24 ساعت و بسامد غذایی بود. شاخص توده بدنی (BMI) نیز محاسبه و با استاندارد CDC2000 مقایسه گردید. نتایج: شاخص نمایۀ تودۀ بدنی و مقایسه آن با استاندارد نشان داد که 9/12% لاغر، 7/12% در معرض خطر افزایش وزن و 7/1% چاق مي‌باشند و شاخص توده بدنی با بعد خانوار نیز ارتباط معناداری دارد. ميزان مصرف شير در 4/35% افراد بين 3-2 بار در هفته مي‌باشد و 8/71% از دانش‌آموزان، ميان وعده در مدرسه مصرف مي‌كردند و تقريباً نيمي از دانش‌آموزان (8/56%) صبحانه مصرف می‌کنند. میزان دریافت کلسیم نسبت‌به استاندارد کمتر مي‌باشد. نتيجه‌گيري: نتايج نشان داد در افراد مورد پژوهش سوء تغذیه وجود دارد. بنابراین لزوم پايش و ارائه خدمات تغذيه‌اي براي اين گروه سني به‌صورت فردي و گروهي توصيه مي‌شود.

Title: بررسي تأثير آموزش بر دانش و مهارت پرستاران در محاسبه دوز داروها

http://knh.shmu.ac.ir/index.php/site/article/view/64

مقدمه: خطاهای دارویی یکی از مهمترین و کشنده‌ترین عوارض درمان محسوب می‌گردند. سالانه هزاران مورد خطای دارویی در بین پرسنل پزشکی و پیراپزشکی گزارش می‌شود. اين مطالعه با هدف تعیین تأثیر آموزش مداوم بر ارتقاء دانش و مهارت پرستاران در محاسبه دوز داروها انجام شده است. مواد و روش‌ها: این پژوهش به شيوه مطالعه چهار گروهي سولمون انجام شده است. نمونه‌های اوليه این پژوهش شامل 100 نفر بود (50 نفر گروه آزمايش و 50 نفر گروه شاهد) که به روش کاملاً تصادفی براساس شماره نظام پرستاری از میان کلیه پرستارانی انتخاب شدند که به فراخوان آموزشی ازطریق سایت دانشگاه و اطلاعیه نصب شده در بخش‌ها پاسخ دادند و دارای معیار ورود به پژ‍‍‍‍‍‍‍وهش نیز بودند. نوع مداخله انجام شده به‌‌صورت اجرای برنامه آموزشی براساس نیازهای آموزشی پرستاران و به‌صورت کارگاهی و انجام کارعملی بود. نتايج: نتايج بعد از آموزش نشان داد كه ميانگين نمره دانش پرستاران در خرده آزمون مربوط‌به داروهاي تزريقي (001/0=P)، حل كردني (001/0P<)، تعيين غلظت دارو (001/0P<) و انفوزيون سرم (007/0=P) به شكل معناداري افزايش يافته است. همچنين اختلاف آماري معناداري بين ميانگين نمرات كسب شده پرستاران در چهار زير گروه روش سولمون وجود داشت (001/0P<) كه تفاوت موجود بين ميانگين نمرات كسب شده گروه اصلی پرستاران آموزش ديده در مقایسه با سایر گروه‌ها بود. نتيجه‌گيري: نتايج نشان‌دهنده تأثير آموزش بر دانش و مهارت پرستاران در محاسبه دوز داروها بود. به‌طوری‌که پرستاران دارای نیازهای عمیق آموزشی درزمینه محاسبه دوز داروها به‌ويژه داروهاي تزريقي بودند. نتايج اين تحقيق در راستاي پژوهش‌هاي مشابه در سرتاسر دنيا، مؤيد آن است كه براي ارتقاي كيفيت مراقبت‌هاي پرستاري، آموزش مداوم پرستاران ضروري است.

Title: نقش ترکیب سنی خانوار در شیوع ناامنی غذایی: یک مطالعه مقطعی در شمال ‌غرب کشور

http://knh.shmu.ac.ir/index.php/site/article/view/62

مقدمه: غذا از جمله نیازهای اساسی بشر است، که تأمین آن در مقوله امنیت غذایی نهفته است. بدون شک درآمد خانوار از مهمترین عوامل در امنیت غذایی خانوار است. ترکیب سنی خانوار نیز ممکن است با تأثیر بر وضعیت اقتصادی خانواده در امنیت غذایی خانوار مؤثر باشد. لذا هدف از این مطالعه تعیین نقش ترکیب سنی خانوار در امنیت غذایی خانوار مي‌باشد. مواد و روش‌ها: این مطالعه به‌روش مقطعی- تحلیلی در دهستان قره‌سوی شهرستان خوی انجام شد. از 3700 خانوار منطقه، 2500 خانوار وارد مطالعه شدند. برای سنجش امنیت غذایی خانوار، از پرسش‌نامه 6 گویه‌ای USDA استفاده شد و داده‌ها‌ی مربوط‌به گروه‌های سنی و درآمد خانوار با استفاده از پرسش‌نامه اطلاعات عمومی جمع‌آوری شد. برای تجزيه‌و‌تحليل از آزمون‌های کای- دو و رگرسیون لجستیک استفاده شد. نتایج: از 2439 خانوار شركت‌كننده در مطالعه، 991 (6/40%) خانوار امن و 1448 (4/59%) خانوار ناامن بودند. بین ترکیب سنی خانوار و وضعیت امنیت غذایی خانوار، رابطه آماری معنادار وجود داشت. همچنین با افزایش بعد خانوار، شانس ناامنی غذایی خانوار کاهش می‌یابد. مقايسه میانگین درآمد سرانه برحسب ترکیب سنی خانوار متفاوت گزارش شده است. شانس ناامنی غذایی در خانوارهای دارای خردسال، بیشتر می‌باشد (05/0P<). ولي افزایش تعداد خردسال و تعداد سالمند، تأثیری بر ناامنی غذایی خانوار ندارد. نتیجه‌گیری: شیوع ناامنی غذایی خانوار در منطقه تحت مطالعه بالا می‌باشد. اقامت سنتی خانوارهای تازه تشکیل شده در کنار پدر و مادر در مناطق روستایی و آموزش تغذیه صحیح به‌ویژه برای خانوارهای دارای خردسال، برای کاهش ناامنی غذایی خانوار پیشنهاد می‌شود.

Title: مقايسه تمرين‌هاي هوازي، مقاومتي و ترکيبي بر نيمرخ ليپيدي و آديپونکتين در مردان غير‌فعال

http://knh.shmu.ac.ir/index.php/site/article/view/116

مقدمه: به تازگی آديپونکتين به‌عنوان يکي از تنظيم گران متابوليسم چربي مطرح شده است. اما پاسخ آن‌ها به فعاليت بدني هنوز به‌درستي معلوم نيست. هدف اين مطالعه تعيين اثر نوع ورزش بر نيمرخ ليپيد و آديپونکتين در مردان غير‌فعال مي‌باشد. مواد و روش‌ها: در اين مطالعه نيمه‌تجربي تعداد 30 آزمودني مرد به‌طور تصادفي در سه گروه فعاليت هوازي (30 دقيقه فعاليت با 60 تا 70 درصد حداکثر اکسيژن مصرفي)، مقاومتي (سه ست با 10 تکرار در هر حرکت با شدت 70 درصد يک تکرار بيشينه) و ترکيبي (فعاليت هوازي به‌مدت 20 دقيقه و فعاليت مقاومتي براي 2 ست) قرار گرفتند و نيمرخ ليپيد و آديپونکتين قبل و 24 ساعت پس از فعاليت در آن‌ها اندازه‌گيري شد. نتايج: نوع فعاليت بر پاسخ کوتاه مدت ليپيد مؤثر بوده اما بر آديپونکتين تأثيري نداشته است. غلظت‌هاي کلسترول تام و HDL-C در گروه مقاومتي 24 ساعت پس از فعاليت در مقايسه با مقادير پايه کاهش معناداري نشان دادند. علاوه‌بر‌اين، غلظت LDL-C نيز کاهش داشت اما اين تغيير معنادار نبود. نوع تمرين بر پاسخ کوتاه مدت TG بي‌تأثير بود. اما زماني که داده‌هاي گروه‌ها با يکديگر ترکيب شدند، کاهش معناداري در غلظت TG مشاهده شد (05/0>P). نتيجه‌گيري: براساس نتايج داده‌ها مشخص شد پاسخ کوتاه مدت آديپونکتين مستقل از نوع فعاليت است، اما فعاليت مقاومتي در مقايسه با فعاليت هوازي و ترکيبي اثر مثبت بيشتري بر تغييرات نيمرخ ليپيد سرم دارد.

Title: بررسی شیوع و برخي عوامل مؤثر بر کمبود کلسیم در زنان باردار و نوزادان آن‌ها

http://knh.shmu.ac.ir/index.php/site/article/view/117

مقدمه: كلسيم یکی از مواد مغذي مهم در دوران بارداري بوده و کمبود كلسيم و ویتامین D عواقب جبران‌ناپذيري بر رشد و توسعه استخوان‌هاي جنين و سلامت زنان باردار دارند. هدف از پژوهش حاضر، بررسی سطح سرمی كلسيم و متابولیت ویتامین D و برخي عوامل مؤثر بر آن در زنان باردار و نوزادان آن‌ها در شهر شاهرود می‌باشد. مواد و روش‌ها: پژوهش حاضر از نوع مقطعی بوده که بر روی 284 نفر از زنان باردار مراجعه‌کننده به بیمارستان فاطمیه شاهرود انجام شده است. در این مطالعه علاوه بر جمع‌آوری اطلاعات دموگرافیک، خون‌گيري از مادران و بند ناف نوزادان جهت اندازه‌گیری كلسيم، 25 هيدروكسي ويتامين D، فسفر و آلكالين فسفاتاز (ALP) انجام گردید. داده‌ها با استفاده از آزمون‌های آماری توصیفی و همبستگی تجزیه‌و‌تحلیل شدند. سطح معناداری در تمام آزمون‌ها 05/0 در نظر گرفته شد. نتایج: ميانگين سن زنان 3/5±6/26 سال بود. در 5/33% (95 نفر) مادران و 25% (71 نفر) نوزادان كمبود كلسيم مشاهده شد. بين سطح كلسيم مادران و بندناف نوزادان ارتباط آماری مثبت و معناداري وجود داشت (001/0P<، 25/0r=). ارتباط معناداری بين سطح سرمي كلسيم مادران باردار با سن آنان (69/0P=)، وزن نوزاد حین تولد (67/0P=)، تعداد حاملگي (35/0P=) و سطح سرمي ويتامينD  (23/0P=) مشاهده نشد. نتیجه‌گیری: باتوجه به شیوع بالای کمبود کلسیم و ویتامین D در این مطالعه و اثرات مضر آن بر سلامت زنان باردار و نوزادان آن‌ها، پیشنهاد می‌شود علاوه‌بر استفاده از مکمل‌های کلسیم و ویتامین D در زنان باردار، آموزش‌های بهداشتی مناسب نیز در راستای ترویج تغذیه مناسب و افزایش تماس با آفتاب ارائه گردد.

Title: اثر فلونیکسین بر سرطان تخمدان القاء شده توسط 12-7 دی-‌میتل‌بنزاآنتراسن (DMBA) در موش صحرایی ماده نژاد ویستار

http://knh.shmu.ac.ir/index.php/site/article/view/123

مقدمه: سرطان تخمدان یکی از شایع‌‌ترین سرطان‌های موجود در زنان می‌باشد. با‌توجه‌به نقش آنزیم‌های سیکلواکسیژناز (COX) و تولید پروستاگلندین نوع E2 در ایجاد ضایعات توموری، به‌کارگیری ترکیباتی به‌عنوان مهارکننده‌های سیکلواکسیژناز در ممانعت از ایجاد سرطان می‌تواند مؤثر باشد. پژوهش حاضر با هدف ارزیابی نقش فلونیکسین به‌عنوان مهارکننده غیر‌انتخابی آنزیم سیکلواکسیژناز در بروز سرطان تخمدان برروی موش‌های صحرایی ماده نژاد ویستار انجام شده است. مواد و روش‌ها: در این مطالعه تجربی از 48 سر موش صحرایی ماده نژاد ویستار در 6 گروه استفاده شد. به‌منظور القای سرطان از تزریق مستقیم 7-12 دی‌-میتل‌بنزاآنتراسن (7,12 Dimethyl Benza Anthracene) به داخل تخمدان استفاده شد. گروه‌های مورد آزمایش شامل گروه شاهد منفی (بدون تزریق)، گروه شاهد با تزریق DMBA، گروه شاهد مثبت همراه با تزریق سالین (Saline+DMBA) و 3 گروه مداخله که بعد از ایجاد تومور توسط DMBA از روز دهم به بعد به‌طور 2 روز در میان، مورد تزریق عضلانی فلونیکسین مگلوماین در دوزهای 5/1، 1، 5/. میلی‌گرم قرار گرفتند. مقایسه بین گروه‌ها با استفاده از آزمون آنالیز واریانس یک‌طرفه انجام شد. نتايج: میانگین وزن تومور در گروه‌های دریافت‌کننده دارو در مقایسه با گروه‌های شاهد مثبت و DMBA به‌طور معناداری کاهش یافته است. میانگین وزن تخمدان در سه گروه دریافت‌کننده فلونیکسین نسبت‌به گروه شاهد منفی (نرمال) اختلاف آماری معناداری را نشان نمی‌دهد (05/0P>). نتیجه‌گیری: این تحقیق تأثیر مثبت فلونیکسین در درمان سرطان تخمدان را تأیید می‌کند. به‌طوری‌که برش‌های میکروسکوپی حاصل از بافت تخمدان در گروه‌های دریافت‌کننده دارو نشان می‌دهد، سلول‌های سرطانی در حال تحلیل رفتن هستند تا جایی‌که سلول‌ها مجدداً در حال به‌دست آوردن نظم طبیعی خود می‌باشند.
